# Callous-Unemotional Traits, Harm-Effect Moral Reasoning, and Bullying Among Swedish Children

**DOI:** 10.1007/s10566-017-9395-0

**Published:** 2017-03-09

**Authors:** Robert Thornberg, Tomas Jungert

**Affiliations:** 1 Department of Behavioural Sciences and Learning, Linköping University, 58183 Linköping, Sweden; 2 Department of Psychology, Lund University, Lund, Sweden

**Keywords:** Psychopathy, Callous-unemotional traits, Moral reasoning, Bullying

## Abstract

**Background:**

Although callous-unemotional (CU) traits have been associated with bullying among children and adolescents, relatively little is known about whether each of the three sub-constructs of CU traits—callous, uncaring, and unemotional—are associated with bullying when they are considered concurrently in the analysis.

**Objective:**

This study was the first to examine in a single model whether callous, uncaring, and unemotional traits are directly related to the perpetration of bullying and to harm-effect moral reasoning in bullying among children as well as whether these three CU traits are indirectly related to bullying mediated by harm-effect moral reasoning.

**Methods:**

Self-reported data on CU traits, harm-effect moral reasoning in bullying situations, and bullying perpetration were collected from 381 children from 13 schools in Sweden. Structural equation modeling was used to test the hypotheses.

**Results:**

When all three sub-constructs of CU traits were included in a single model, greater callousness and uncaring were directly associated with greater bullying. In contrast, greater harm-effect moral reasoning was associated with less bullying. Moreover, greater callousness and unemotional were indirectly associated with greater bullying through the reduced use of harm-effect moral reasoning.

**Conclusions:**

Our findings demonstrate that all three CU traits are important to address, although their associations with bullying took some different paths, and that callousness appears to be the most important CU trait in relation to bullying.

## Introduction

School bullying is commonly defined as repeated aggression directed at target individuals who are disadvantaged or less powerful in their interactions with the bully or bullies (Borntrager et al. 2009; Malecki et al. 2015; Olweus 1993). The Swedish National Agency for Education (2014) defines bullying (“mobbning”) as a form of offensive treatment or harassment in terms of a repeated negative act in which one or more people consciously and with intention inflict or try to inflict injury or nuisance on someone. According to a recent report from the Swedish National Agency for Education (2016), nine percent of the Grade 4–6 students (around 10–12 years-old) and three percent of the Grade 7–9 students (around 13–15 years-old) reported being bullied by other students on a weekly basis.

The Swedish Education Act (Skollagen, 2010:800, Chapter 6) states that all forms of offensive treatment that violate a student’s dignity are forbidden in school, and the school staff must act and investigate every suspicion or identified case of offensive treatment in school. The Swedish National Agency for Education (2014) has published recommendations on how to counteract discrimination, harassment, and other offensive treatment, including bullying. The schools should regularly survey and analyze students’ sense of school safety and the prevalence of all forms of offensive treatment. There should be clear routines for how to investigate, intervene in, and document all suspected cases of bullying. A continuous and informal values education embedded in everyday school life (rather than as classroom lessons) and based on basic democratic values and human rights is recommended as a part of bullying prevention. A positive school climate is emphasized, and the school staff should be present and oversee spaces in which students are present. Interventions should start with an all-embracing investigation that includes an analysis of the causes of bullying and should include both the perpetrator and the victim. Parents of the involved students have to be informed as soon as possible. In every single case the school principal should also consider whether or not to report the incident to other authorities (the social services, the police, or the Swedish work environment authority). The schools should have procedures for managing acute situations, and all interventions should be documented, followed up, and evaluated. The Swedish National Agency for Education (2014) does not recommend any particular anti-bullying program.

Bullying is associated with well-known negative effects on the health and psychosocial development of the victims, including poor self-esteem, depression, anxiety, suicidal thoughts, suicidal behavior, social isolation, further victimization, and poor health (for meta-analyses, see Gini and Pozzoli 2013; Reijntjes et al. 2010). Perpetrating bullying behavior is in turn a predictor of delinquency, violence, criminality, and other antisocial behaviors in later adolescence and adulthood (e.g., Chan and Chui 2013; for meta-analyses, see Ttofi et al. 2011, 2012). Thus, not only victims but bullies too are at risk of psychological and social maladjustments. Although bullying is produced by a complex interplay between individual and contextual factors, the focus in this project was on individual factors associated with the perpetration of bullying. More specifically, the purpose of the current study was to examine the relations between harm-effect moral reasoning, psychopathic (callous-unemotional) traits, and bullying behavior among children.

### Harm-Effect Moral Reasoning

According to social domain theory (Nucci 2001; Turiel 2008), children and adolescents develop and construct their social knowledge in different domains through their social experiences. These moral domain structures are developed in the long-term memory through repeated experiences of social interactions that share the core features of “actions that cause others harm”. If activated, these latent mental structures influence how children perceive, evaluate, and behave in various social situations (Arsenio and Lemerise 2004). From early preschool years, children distinguish between morality (i.e., concepts such as human welfare, justice, and rights as well as the regulation of actions that affect others in these terms) and social convention (i.e., social norms that regulate actions with no inherently harmful effects on other people). They judge moral transgressions as wrong regardless of whether rules exist and as more serious and worse than conventional transgressions, and they tend to justify such judgments in terms of the harm or unfairness that these actions cause (for a review, see Nucci 2001).

In accordance with social domain theory, the vast majority of early adolescents consider bullying to be highly immoral and as wrong regardless of any rules against bullying and as more wrong than conventional transgressions. They justify these judgments by referring to the harm that bullying causes (Thornberg 2010; Thornberg et al. 2016). A crucial part of such judgments of moral transgressions like bullying is the link between the concept of the harm that the action causes and the aroused moral emotions such as empathy, sympathy for the victims, transgressive guilt, guilt for inaction as a bystander, or moral anger toward the perpetrators (Hoffman 2000). In normal moral functioning, such ‘hot’ affective content is associated with the construction of moral-action schemas and has been integrated within the overall conceptual framework guiding the child’s morality (Hoffman 2000; Nucci 2001). As Blair et al. (2001) put it, “the importance of responsiveness to distress cues for the emergence of morality can be seen from the work on the moral/conventional distinction” (p. 800). In accordance with this, research has revealed that bullies have lower levels of affective empathy (Caravita, Blasio, and Salmivalli 2009; Jolliffe and Farrington 2006; Muñoz et al. 2011) and tend to attribute social-conventional characteristics to moral issues more than their peers do (Caravita, Miragoli, and Di Blasio 2009). Therefore, we hypothesized that the degree of harm-effect moral reasoning triggered by bullying scenarios is negatively associated with bullying among children. In other words, we assumed harm-effect moral reasoning to be a protective factor against bullying behavior.

### Callous-Unemotional Traits

Psychopathy can be understood as a severe deficit in human conscience (Frick and White 2008; White and Frick 2010) and essentially as a moral disorder (Blair 2007). Callous-unemotional (CU) traits are prominent in most conceptualizations of psychopathy (Frick and White 2008; White and Frick 2010). They refer to “a specific affective (absence of guilt, constricted display of emotion) and interpersonal (failure to show empathy, callous use of others for one’s own gain) style” (Fanti et al. 2009, p. 285). CU traits include a lack of concern for the feelings of others, a lack of remorse, a lack of concern for one’s performance in important activities, and superficial or shallow expressions of emotions (Frick et al. 2014; Kimonis et al. 2015). Psychopathic traits in general, and CU traits specifically, are related to severe conduct problems and delinquency (Frick and White 2008; Frick et al. 2014), and CU traits among children and adolescents are positively associated with aggression (Ansel et al. 2015; Fanti et al. 2009, 2013; Kimonis et al. 2008, 2015; Stickle et al. 2009; Thornton et al. 2013), including bullying (Ciucci et al. 2014; Crapanzano et al. 2011; Fanti et al. 2012; Fanti and Kimonis 2012; Golmaryami et al. 2016; Kimonis et al. 2015; Muñoz et al. 2011; Thornton et al. 2013).

CU traits have also been found to be associated with the acceptance of aggressive responses in social situations (Stickle et al. 2009). When judging hypothetical moral transgression scenarios, early adolescents with conduct problems and high levels of psychopathic traits tend to be less likely to refer to others’ welfare in their justifications (Blair et al. 2001) and are more likely to allow transgressions if there are no rules prohibiting these transgressions (Blair 1997; Blair et al. 2001) compared to their peers with conduct problems but low levels of psychopathic traits. In their study, Pardini and Byrd (2012) found that children with greater CU traits were less likely to expect that aggression would result in the victim suffering and were less likely to have feelings of remorse. They were also less concerned about the victim’s suffering following acts of aggression. Furthermore, children with greater CU traits reported less empathic concern and sadness in response to others’ distress. A number of studies have consistently shown a negative association between CU traits and other measures of empathy, especially affective empathy (for a review, see Frick et al. 2014). Therefore, we hypothesized that greater CU traits are associated with less harm-effect moral reasoning in bullying situations.

CU traits are multidimensional constructs that consist of the following three sub-constructs: (a) *callousness*, that is, a lack of empathy with and concerns about others’ welfare, harm, or suffering, (b) *uncaring*, that is, a lack of concern about one’s performance in socially important activities, and (c) *unemotional*, that is, not being open about and expressing or showing one’s feelings (Essau et al. 2006; Fanti et al. 2009; Kimonis et al. 2008). Nevertheless, most previous research has only investigated the association between a global index of CU traits and variables such as aggression and bullying, although there are a few exceptions. Kimonis et al. (2008) found callousness to be more consistently correlated with aggression than uncaring, and unemotional was almost not correlated with aggression at all. In regression analyses conducted by Fanti et al. (2009), callousness and uncaring, but not unemotional, were related to bullying, and only callousness was related to proactive aggression. In addition, Muñoz et al. (2011) reported that uncaring and callousness, but not unemotional, were positively correlated with both direct and indirect bullying. Ciucci et al. (2014) found that callousness and uncaring, but not unemotional, were correlated with bullying. Finally, Ansel et al. (2015) found callousness and uncaring, but not unemotional, to be correlated with aggression. With reference to these studies, we hypothesized that the traits of callousness and uncaring, but not unemotional, are associated with bullying.

Previous research has shown how a global index of CU traits is associated with the acceptance of aggressive responses in social situations. However, it is currently unknown whether the three sub-constructs of CU traits are directly associated with harm-effect moral reasoning in bullying situations. It is also unknown whether they are indirectly associated with bullying mediated by harm-effect moral reasoning. In our work, callousness represents a lack of empathy with and concern about others’ welfare, harm, or suffering; uncaring represents a lack of concern about school work, task performance, or work achievement; and unemotional is not being open about and showing one’s feelings. Therefore, we assumed that among the three sub-constructs of CU traits only callousness would be related to less harm-effect moral reasoning in bullying and that it would be at least partly indirectly associated with bullying through harm-effect moral reasoning.

### Aim and Hypotheses

The aim of the present study was to examine—within a single model—whether callous, uncaring, and unemotional traits are directly related to harm-effect moral reasoning and bullying as well as if they are indirectly related to bullying mediated by harm-effect moral reasoning among children. Based on previous research, we hypothesized that greater callousness and uncaring is directly associated with greater bullying and that unemotional is unrelated to bullying. We also hypothesized that greater harm-effect moral reasoning is directly associated with less bullying. We further hypothesized that callousness contributes to explaining the variance of harm-effect moral reasoning (greater callousness is associated with less harm-effect moral reasoning), and thus would, at least partly, be indirectly related to bullying mediated by harm-effect moral reasoning.

## Methods

### Participants and Procedure

A cross-sectional design and structural equation modeling were used to test our hypotheses. Participants were recruited from 13 elementary schools in Sweden. A non-probability, two-step sampling was used in the study. First, a purposive sampling of schools was carried out, which resulted in the inclusion of 13 schools, including two schools in the countryside, one school in a small town, nine schools in different neighborhoods within two medium-sized Swedish cities, and one school in a large Swedish city. Second, a convenience sampling was conducted in each school based on cooperation with class teachers and limited to Grades 5 and 6. The initial sample consisted of 458 children. Parental consent letters were distributed to all of the families, and informed consent was required from all individual participants included in the study as well as from their parents. Twenty-four children did not participate either because they did not want to or because they did not obtain parental consent, 32 children did not participate because they were absent due to sickness during the data collection, and 21 children were excluded from the analyses because they did not fill in their questionnaires after having answered the first few items. Because we could not know whether additional data were missing at random (MAR) or missing not at random (MNAR), we handled the missing data by applying the expectation maximization (EM), which is available in EQS. The EM technique is recommended when the data are MNAR or when it is not possible to know if the data are MAR (see e.g. Myers 2011; Roth et al. 1999). Thus, the final sample consisted of 381 children (198 boys and 183 girls; age range = 10.0–13.5 years, *M* = 12.0 years, *SD* = .73 years), resulting in a participation rate of 83.2%. This two-step sampling procedure led to a sample of children from different socioeconomic (from lower to upper middle class) and socio-geographic backgrounds. The questionnaire was filled out by the participants in their ordinary classroom settings. The study received ethical approval from the Regional Ethical Review Board at Linköping.

### Measures

The questionnaire was adopted from Thornberg et al. (2016). The first page of the questionnaire began with this introduction, “This questionnaire is about a school called Aspen Grove School, and it is like your own school. There are many rules at Aspen Grove School. Here are some examples of rules that Aspen Grove School has: (1) be quiet in the classroom during deskwork, (2) don’t swear when talking, (3) don’t wear a cap in class, (4) don’t beat or kick others, (5) don’t spread lies or rumors about one another, (6) don’t ostracize anyone, (7) don’t tease one another, (8) don’t speak in the classroom when the teacher is talking to the class, and (9) don’t say no to children who want to join in the football game during recess”.

This list of school rules was followed by a general statement and a few instructions, “Now the teachers at Aspen Grove School have decided to take away some rules at the school. In this questionnaire, we ask you what you think about this. When you answer, try to ignore what the teachers or other adults at your school think. We want to know what you as a student think”. Twelve vignettes (hypothetical scenarios) then followed. These vignettes represented eight prototypical examples of bullying (repeated moral transgressions) and four repeated conventional transgressions in school settings. The common structure of each story was that it began with the teachers at the fictional school telling the students that they had repealed a specific school rule. After that, the story described an incident in which one or more students engaged in one of these previously forbidden acts. Four vignettes described direct bullying (two for physical bullying and two for verbal bullying), four vignettes described relational bullying (two for negative rumor-spreading and two for ostracizing), and four vignettes described repeated conventional transgressions. Only the eight bullying vignettes were used in the current analysis. Here is an example of a physical bullying vignette from the questionnaire:

The teachers told the students that they have taken away the rule about not beating one another. Over the next few months, some students are beating the same student during recess several times a week.

#### Harm-Effect Moral Reasoning in Bullying

The participants were asked to judge the actual behavior of the transgressor(s) after reading each vignette. In order to assess their justifications for their judgments regarding the various transgression, the students were asked an open question to provide reasons for each judgment, ‘Why do you think so? I think so __________’ (followed by four or five blank lines). Two raters worked together to code the reasons and justifications according to the coding scheme used in Thornberg (2010), which consists of moral, structuring, protecting, indifference, socio-normative, personal choice, pleasure, impulse, and “other” reasons. Disagreements were discussed until a consensus was reached, and the negotiated consensus was then coded. For the analysis in this paper, only the presence of harm-effect moral reasoning, that is, judging that the transgression was wrong by referring to the harm it causes others (e.g., “Because it’s unfair. The one who is being beaten up might be scared and feel unsafe in school,” “Because even though there is no rule against it, it would still hurt other people,” “Because if someone did that to you, you would be sad,” and “Because the other guy is harmed”). A global index of moral reasoning was calculated by using the sum of the presence of moral reasoning across the eight bullying vignettes (i.e., eight dichotomous variables), and was therefore constructed as a nine-point scale from 0 to 8 (α = .80).

#### Callous-Unemotional Traits

The questionnaire used in this study included the Swedish version of the Inventory of Callous-Unemotional Traits (ICU; Frick 2004). The ICU is a 24-item self-report scale used to assess CU traits in youth, and it is rated on a four-point scale (0 = “Not at all true”, 1 = “Somewhat true”, 2 = “Very true”, and 3 = “Definitely true”). The ICU was translated from English into Swedish by two master students in psychology at the end of their training and then back translated into English by a former master student in sociology. With reference to previous validation studies identifying and confirming the three factors in the ICU (callousness, uncaring, and unemotional; Ciucci et al. 2014; Essau et al. 2006; Fanti et al. 2009; Kimonis et al. 2008; Roose et al. 2010), we also performed an exploratory principal component factor analysis using the Maximum Likelihood algorithm and the Direct Oblimin rotation with three fixed factors to examine the expected factors in the Swedish version. Several items had to be dropped from further analysis due to cross loadings. In the final model, four items loaded on the first factor, *callousness* (items 4, 12, 18, and 21; α = .83), three items loaded on the second factor, *uncaring* (items 3, 15, and 23; α = .66), and five items loaded on the third factor, *unemotional* (items 1, 6, 14, 19, and 22; α = .77). These three factors had low to moderate correlations (*r* = .01–.26). KMO was .77, which indicated a good structure of factors. The general index of CU traits based on the three factors and their items had an acceptable internal reliability (in total 12 items; α = .72).

#### Bullying

The Swedish version (Olweus 1996a) of the Revised Olweus Bully/Victim Questionnaire (OBVQ; Olweus 1996b) contains a definition of bullying and a set of questions and was used as part of the questionnaire in this study to measure the levels of bullying perpetration. In the current study, we used the behavioral items after the global question of bullying another student(s), and we excluded the racist bullying item and the sexual bullying item because the first one was assumed to be, at least partly, a function of neighborhood (and thus, vulnerable to contextual bias in terms of variation in ethnic composition across different neighborhoods) and the second was considered age-inappropriate. In total, seven items from the questionnaire were analyzed (α = .85). However, because the Lagrange test indicated that the first two behavioral items in the bullying scale should instead be added to the callousness scale when bullying was included in the same model as callousness, we also dropped these two bullying items in the current study (see the SEM section in the findings below). The five remaining bullying items in the OBVQ still covered physical, verbal, relational, and cyber bullying and had good internal reliability (α = .81).

## Results

### Confirmatory Factor Analysis

In order to test whether the data fit a three-factor model of CU traits, we performed a confirmatory factor analysis with the EQS 6 program (Bentler 1995). Model estimation was performed within the framework of structural equation modeling (SEM) using the full information maximum likelihood estimation (Little and Rubin 2002). Model fit was evaluated by several commonly used fit indices within SEM, including the Chi square statistic on degrees of freedom (χ^2^/df), the comparative fit index (CFI), and the Root Mean Square Error of Approximation (RMSEA) with a 90% confidence interval. The three statistics indicated an acceptable fit (N = 381; CFI = .92, χ^2^/df = 151.20/51 = 2.96, *p* < .001, RMSEA = .07; CI 90% [.06, .09]). Table 1 shows the factor loadings of the standardized solution and how indicators F1 through F3, which are synonymous with these three factors, were computed.

**Table 1:** Factor loadings for the best fitting three factor model (N = 381)

Items	F1	F2	F3
4. I do not care who I hurt to get what I want	.73		
12. I seem very cold and uncaring to others	.81		
18. I do not feel remorseful when I do something wrong	.68		
21. The feelings of others are unimportant to me	.75		
3. I care about how well I do at school or work^a^		.44	
15. I always try my best^a^		.86	
23. I work hard on everything I do^a^		.64	
1. I express my feelings openly^a^			.75
6. I do not show my emotions to others			.59
14. It is easy for others to tell how I am feeling^a^			.59
19. I am very expressive and emotional^a^			.68
22. I hide my feelings from others			.56

*F1* callous, *F2* uncaring, *F3* unemotional

^a^Reverse scored items; *r*(F1, F2) = .26***, *r*(F1, F3) = .01, *r*(F2, F3) = .13*

### Descriptive Statistics and Intercorrelations

Means, standard deviations, and bivariate correlation coefficients of all study variables are reported in Table 2. Because age did not correlate significantly with any other variable, it was excluded from the table. Boys were more prone to bullying and more likely to display general CU traits and all three sub-constructs (although uncaring and unemotional were only very weakly associated with gender), whereas girls were more prone to display harm-effect moral reasoning. Within the construct of CU traits, callousness and uncaring correlated significantly with each other, but unemotional was only weakly related to uncaring. Nevertheless, the general CU traits index was moderately correlated with all three sub-constructs of CU traits. Moreover, greater levels of callousness, uncaring, and unemotional (as well as greater general CU traits) were associated with lower harm-effect moral reasoning. Callousness was, however, more strongly linked to harm-effect moral reasoning compared with the two other sub-constructs of CU traits. In addition, greater levels of callousness, uncaring, and unemotional (as well as greater general CU traits) were associated with greater bullying, although the link between unemotional and bullying was significantly weaker compared with the two other correlations. Finally, greater harm-effect moral reasoning was associated with less bullying.

**Table 2:** Descriptive statistics and bivariate correlations among study variables

	M	SD	1	2	3	4	5	6	7
1. Gender	49% female		1						
2. Callousness	.36	.62	−.24***	1					
3. Uncaring	.70	.59	−.11*	26***	1				
4. Unemotional	1.42	.57	−.11*	.01	.13*	1			
5. CU general	.89	.38	−.24***	.64***	.61***	.67***	1		
6. Harm-effect moral reasoning	5.60	2.34	.18**	−.32***	−.14**	−.17**	−.33***	1	
7. Bullying	.12	.32	−.16**	.33***	.25***	.12*	.35***	−.34***	1

The bullying variable consists of the five remaining items * *p* < .05; ** *p* < .01; *** *p* < .001

### SEM

**Fig. 1:**
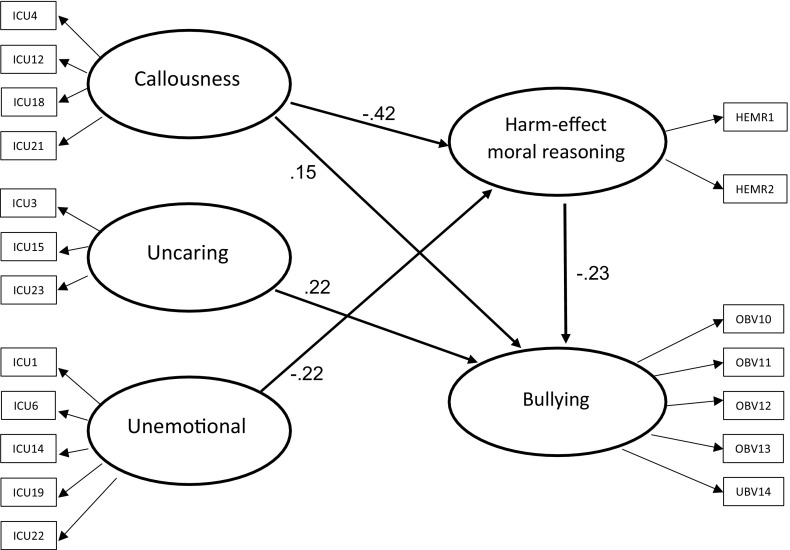
The best-fitting model for the whole sample. All path coefficients are significant at *p* < .05

When we attempted to confirm the hypothesized model, the fit statistics for the model were not acceptable (N = 381; CFI = .71; χ^2^/df = 1091.75/160 = 6.82, *p* < .001, RMSEA = .13; CI 90% [.12, .13]). Indeed, the Wald test suggested that the path between uncaring and harm-effect moral reasoning and the path between unemotional and bullying should be dropped, and the Lagrange multiplier test indicated that two of the bullying items (the first two items in the OBVQ) should be added to the callousness items, which would result in a significant improvement in the model fit. Based on the Wald test, we decided to drop the path between uncaring and harm-effect moral reasoning and the path between unemotional and bullying from the model. Based on the Lagrange test, we decided to drop the two bullying items from the model (as mentioned in the Methods section) because we wanted to test our hypothesized model for the associations between callousness and school bullying. The results of the SEM are shown in Fig. 1, which includes all of the standardized coefficients. The model fit statistics for the model (N = 381; CFI = .92; χ^2^/df = 348.67/145 = 2.4, *p* < .001, RMSEA = .06; CI 90% [.05, .07]) indicated an adequate fit. The Wald test suggested no further modifications of the model, and this allowed us to conclude that the model fit the sample. Figure 1 shows all of the path coefficients in the overall model. Among the direct paths between CU traits and harm-effect moral reasoning, both callousness (−.42) and unemotional (−.22) had significant and negative associations, and among the direct paths between the CU traits and bullying, uncaring (.22) and callousness (.15) had significant and positive associations. There was only an indirect association between unemotional and bullying via harm-effect moral reasoning because the path between harm-effect moral reasoning and bullying was negative and significant (−.23). Thus, the model indicated that (a) callousness and unemotional were directly associated with harm-effect moral reasoning; (b) uncaring and callousness were directly associated with bullying, and (c) unemotional was only indirectly associated with bullying via harm-effect moral reasoning.

### Mediation by Harm-Effect Moral Reasoning

We tested the mediating role of harm-effect moral reasoning with the Preacher and Hayes’ (2008) macro in the SPSS software package, which is designed for testing and comparing indirect effects in multiple mediator models. Separate analyses were conducted for bullying as the dependent variable, and callousness and unemotional were used as the independent variables. Bootstrapping with the number of bootstrap samples set at 5000 was used to calculate 95% confidence intervals for the specific indirect effects of harm-effect moral reasoning. Preacher and Hayes (2008) recommend bootstrapping, especially when testing for mediation, because it does not require normality of the sampling distribution. Bootstrapping furthermore provides distributions for each statistic from which confidence intervals can be derived (Preacher and Hayes 2004).

An initial multiple mediation analysis was run with unemotional as the independent variable, bullying as the dependent variable, and harm-effect moral reasoning as the mediator. The results confirmed the mediating role of harm-effect moral reasoning in the relationship between unemotional and bullying because zero was not contained in the confidence interval (95% CI .01, .07; effect size = .03).

Finally, we ran an analysis to test callousness as the independent variable, harm-effect moral reasoning as the mediator, and bullying as the outcome variable. Zero was not contained in the confidence interval (95% CI .02, .08; effect size = .04), suggesting that harm-effect moral reasoning mediates the relationship between callousness and bullying (Preacher and Hayes 2004, 2008).

## Discussion

This study was the first to examine whether the CU traits of callousness, uncaring, and unemotional were directly related to bullying perpetration and harm-effect moral reasoning in bullying as well as if they were indirectly related to bullying perpetration mediated by harm-effect moral reasoning among children within a single model. CU traits have been associated with bullying and aggression more generally among children and adolescents (e.g., Ansel et al. 2015; Ciucci et al. 2014; Fanti et al. 2012, 2013; Golmaryami et al. 2016; Kimonis et al. 2015; Thornton et al. 2013). Nevertheless, relatively little is known about whether each of the three sub-constructs of CU traits (callousness, uncaring, and unemotional) are associated with bullying when they are considered concurrently in the analysis. There are, however, some important exceptions (Ciucci et al. 2014; Fanti et al. 2009; Muñoz et al. 2011) showing that callousness and uncaring, but not unemotional, appear to contribute to explaining the variation of bullying. In accordance with previous research and our hypothesis, we showed that only callousness and uncaring were directly associated with bullying.

Moreover, previous studies have demonstrated that CU traits as a unidimensional construct are associated with the acceptance of aggressive responses (Stickle et al. 2009), having less concern about victims’ suffering (Pardini and Byrd 2012), and being less likely to make references to others’ welfare when evaluating hypothetical moral transgression scenarios (Blair et al. 2001). However, the current findings revealed that when all three sub-constructs of CU traits were included in a single model, greater levels of callousness and unemotional, but not greater levels of uncaring, were directly associated with less harm-effect moral reasoning when judging bullying behavior. In accordance with our hypothesis, the current findings showed that callousness was both directly related to bullying and indirectly related to bullying mediated by harm-effect moral reasoning. The negative link between callousness and harm-effect moral reasoning was expected because the theoretical construct of callousness represents a lack of concerns about others’ welfare, harm, and suffering (Essau et al. 2006; Fanti et al. 2009; Kimonis et al. 2008). However, in contrast to our hypotheses, we also found that unemotional was indirectly related to bullying via harm-effect moral reasoning. One possible explanation for this might be that unemotional, which refers to a lack of emotional expression (Essau et al. 2006; Fanti et al. 2009; Kimonis et al. 2008), indicates an emotional disengagement or disconnection and thus a possible lack of emotional awareness of oneself and others. In contrast, emotional intelligence is defined as “the ability to perceive, manage, and reason about emotions within oneself and others, and to use this information to guide adaptive thinking and behavior” (Kahn et al. 2016, p. 903). Abe et al. (2013), for example, found that among undergraduate medical students, expressing one’s emotions and listening to others increased emotional intelligence. It would therefore be plausible to assume that children who are not expressing their emotions are less engaged with emotions (are unemotional) and therefore less able to recognize emotions such as sadness and distress in others. This in turn would make them less likely to engage in harm-effect moral reasoning in bullying situations. If they are not aware of negative or distressful emotions of others, they will not consider and reason upon these emotions. They would therefore be less inhibited from engaging in the perpetration of bullying. Hence, callousness and unemotional should together have a greater impact on harm-effect moral reasoning than callousness alone.

## Limitations

This study has a number of limitations. First, the variables in the present study were assessed through self-report, which might have inflated variable associations due to shared method variance. Second, it is important to recognize that investigating how children respond to hypothetical scenarios is not the same as investigating how they would respond in real-life situations. The study can therefore be problematized in terms of ecological validity (cf. Cicourel 1982). Nevertheless, this technique enables researchers to collect responses from all of the participating children with regards to the same situations. Some studies have demonstrated that children judge transgressions in real-life situations similar to those in hypothetical situations (Smetana et al. 1993; Turiel 2008), a finding that addresses the ecological validity concerns. Third, several items from the ICU had to be excluded in order for the three-factor model to approach an adequate fit to the data. However, even though the three-factor model originally suggested by Essau et al. (2006) has been replicated as three-factor, second-order factor, and bifactor models, prior validation studies have had problems achieving an acceptable fit on all indices (e.g., Fanti et al. 2009; Kimonis et al. 2008; Roose et al. 2010). Moreover, whereas a study with young children (aged 7–12 years) resulted in a two-factor model (Houghton et al. 2013), another study including a Dutch version of the ICU ended up with a five-factor model (Feilhauer, Cima, and Arntz 2012). Excluding items and thereby shortening the ICU scale has been crucial to meeting statistical standards and to successfully confirming the three theoretical sub-constructs of the CU traits as they have been represented in the literature (Essau et al. 2006; Fanti et al. 2009; Kimonis et al. 2008). In addition, the reduction of items in the ICU scale resulted in the removal of the overlaps between the constructs of callousness and uncaring that were found in the original scale (e.g., “I do not care about doing things well” and “I do not care about being on time” as callousness items instead of uncaring items, and “I try not to hurt others’ feelings” and “I do things to make others feel good” as reversed uncaring items instead of callousness items in the original scale; see Essau et al. 2006; Fanti et al. 2009; Kimonis et al. 2008). Moreover, only using five behavioral items of the original bullying scale in the OBVQ (Olweus 1996a, b) might threaten the validity of the bullying variable. On the other hand, all five items covered physical, verbal, relational, and cyber bullying and had good internal reliability. Finally, a note of caution needs to be sounded regarding the generalization of the findings because this sample of Swedish children may or may not be similar to the population of children that readers primarily work with or are interested in studying.

## Implications

Frick and White (2008) highlight the importance of designing preventive interventions aimed at addressing the particular characteristics of CU traits, such as promoting the development of empathy and concern for others, even before aggression and conduct problems have become severe enough to warrant a psychiatric diagnosis. Hence, screenings, preventions, and early interventions in nursery and preschool settings are likely to be important. Because parenting style is associated with psychopathic youth (for a review, see Farrington et al. 2010), parental training should be a primary intervention strategy. For example, harsh and inconsistent parenting is associated with more stable patterns of CU traits (Frick et al. 2003; Pardini and Loeber 2008; Waller et al. 2012), while Pardini et al. (2007) reported that whereas harsh parenting was related to increases in CU traits, parental warmth predicted decreases in CU traits in early adolescents over a 1-year period. A positive parent–child relationship from an early preschool age that reflects high degrees of warmth and responsiveness can serve as a protective factor that decreases the probability of antisocial development in children who are at risk due to elevated CU traits (Kochanska et al. 2013). Our findings demonstrated that all three CU traits are important to address, although their associations with bullying took some different paths. Callousness seems to be the most important to identify and reduce because it had the strongest impact on bullying and was both directly associated with bullying and indirectly associated with bullying mediated by harm-effect moral reasoning. Uncaring was directly associated with bullying, and unemotional was indirectly associated with bullying mediated by harm-effect moral reasoning.

The current study showed that not only callousness but also unemotional were negatively related to harm-effect moral reasoning, which in turn was negatively related to bullying. A clear implication of this is that it is important to ensure that anti-bullying programs and practices facilitate, educate, and inculcate harm-effect moral reasoning while at the same time reducing callousness and unemotional states among children. A way of working with this might be to promote children’s emotional engagement and competence as well as their overall empathic concerns for other people, especially for victim suffering (cf., Hoffman 2000). Dadds et al. (2012) reported a randomized controlled trial of “emotional-recognition-training” with children and adolescents with behavioral/emotional problems, and they found that the program produced significant improvements in affective empathy and conduct problems for the participants with high CU traits. Although psychopathic children and adolescents might be potentially problematic in psychotherapy, there are research findings showing that they might also make progress in such treatment settings (Salekin 2010). Nevertheless, as noticed by Chialant et al. (2016), psychotherapy and empathy training might be ineffective and might even result in negative treatment outcomes, with worsening of psychopathy. Training of socio-emotional skills might, for instance, actually increase psychopathic children’s and adolescents’ capacity to harm and manipulate others without receiving attention from adults. A crucial issue then is whether empathy trainings “actually stimulate empathy or merely the mimicking of empathic responding” (p. 279; for a further discussion on psychopathic predatory violence, empathy, and interventions with empathy training, see Chialant et al. 2016).

Because the trait or pattern of being careless or uncaring about one’s duties, performances, and achievements was found to be directly linked with bullying, efforts to identify and reduce such uncaring should be included in bullying prevention and intervention programs. A permissive approach toward uncaring would therefore not be helpful. In contrast, authoritative school discipline (Gregory and Cornell 2009) and an authoritative teacher style (not to be confused with an *authoritarian* teacher style) includes warmth, responsiveness, autonomy-supportiveness, high expectations, demandingness, and fair and consistent rule enforcement (Wentzel 2002). Authoritative school discipline and teacher style are related to both greater academic achievement (Gregory and Weinstein 2004) and less antisocial behavior, aggression, and bullying (Cornell and Huang 2016; Gerlinger, and Wo 2016; Gregory et al. 2010). An authoritative teacher style is also prominent in the core principles behind the Olweus Bullying Prevention Program (Olweus and Limber 2007) and should counteract all three CU traits as well as promote harm-effect moral reasoning among children.
